# Interprofessional work in operating rooms: a qualitative study from Sri Lanka

**DOI:** 10.1186/s12893-016-0177-7

**Published:** 2016-09-05

**Authors:** Vathsala Jayasuriya-Illesinghe, Sepali Guruge, Bawantha Gamage, Sherry Espin

**Affiliations:** 1 Daphne Cockwell School of Nursing, Ryerson University, 350 Victoria St, Toronto, ON M5B 2K3 Canada; 2 Faculty of Medical Sciences, University of Sri Jayewardenepura, Colombo, Sri Lanka

**Keywords:** Interprofessional work, Sri Lanka, Surgical errors, Teamwork

## Abstract

**Background:**

A growing body of research shows links between poor teamwork and preventable surgical errors. Similar work has received little attention in the Global South, and in South Asia, in particular. This paper describes surgeons’ perception of teamwork, team members’ roles, and the team processes in a teaching hospital in Sri Lanka to highlight the nature of interprofessional teamwork and the factors that influence teamwork in this setting.

**Methods:**

Data gathered from interviews with 15 surgeons were analyzed using a conceptual framework for interprofessional teamwork.

**Results:**

Interprofessional teamwork was characterized by low levels of interdependency and integration of work. The demarcation of roles and responsibilities for surgeons, nurses, and anesthetists appeared to be a strong element of interprofessional teamwork in this setting. Various relational factors, such as, professional power, hierarchy, and socialization, as well as contextual factors, such as, patriarchy and gender norms influenced interprofessional collaboration, and created barriers to communication between surgeons and nurses. Junior surgeons derived their understanding of appropriate practices mainly from observing senior surgeons, and there was a lack of formal training opportunities and motivation to develop non-technical skills that could improve interprofessional teamwork in operating rooms.

**Conclusions:**

A more nuanced view of interprofessional teamwork can highlight the different elements of such work suited for each specific setting. Understanding the relational and contextual factors related to and influencing interprofessional socialization and status hierarchies can help improve quality of teamwork, and the training and mentoring of junior members.

## Background

Delivering healthcare requires different professional groups to come together as teams, share information, and reach agreement in their work. However, not all groups of healthcare professionals collaborate effectively as teams. Quality of teamwork among healthcare professionals has been linked to patient mortality, morbidity, and satisfaction with care, as well as healthcare provider job satisfaction [1–4]. Poor quality teamwork is associated with higher rates of medical errors and adverse events for patients [5]. Teamwork within operating rooms have received close attention as they are considered to be highly dynamic environments in which patients are known to be vulnerable to adverse events. Breakdown in information-sharing and lack of or poor communication have been identified as leading to adverse events in operating rooms more so than errors in surgical technique [4–7]. As such, there has been a growing interest in improving quality of teamwork among healthcare professionals, particularly, in surgical settings [8–11]. However, much of the initiatives that have been put in place to improve teamwork have been guided by studies done in developed countries. Much less is known about the quality of teamwork, factors which influence collaboration among healthcare professionals, and ways of improving interprofessional teamwork in low-middle income countries [12, 13].

This paper describes surgeons’ perception of teamwork, team members’ roles, and the team processes in operating rooms in a teaching hospital in the capital province of Sri Lanka, a South Asian setting. The aim of this paper is to describe the nature of interprofessional work and the factors that influence teamwork in this setting.

## Methods

Approval was obtained from the research ethics boards in the institutions with which the authors are affiliated with and the hospital where the study was conducted. The study setting was a teaching hospital in the capital province of Sri Lanka, which provides services to a large urban population and also functions as the clinical training hospital for medical and nursing graduates from a number of universities in the area.^1^

We invited surgeons to volunteer for the study and a female research assistant (RA)-a psychology graduate with prior training and experience in similar research-purposefully selected a sample to represent the different levels of seniority among them. She invited them to participate in an interview at a convenient time. After obtaining verbal consent, the RA conducted individual interviews with selected surgeons in private.

The interviews were semi-structured and lasted 45 to 90 min. The interview guide was first developed based on related literature and the information gathered from informal interviews with surgeons and nurses. The interview guide was revised after a pilot test among a small sample of surgeons separate from those participating in the study. The interviews focused on the usual arrangement of operating room work, and what each member of the team does in the operating room. Follow-up and probe questions were used to explore topics and elicit further details, while at the same time allowing respondents flexibility to discuss issues that were important to them. Questions were modified as new information and/or issues emerged. Participants were given a small honorarium for the time spent on the study. The interviews were concluded once data saturation occurred after gathering information from 15 surgeons.

The participants were given the option of conducting the interview in the local languages (Sinhalese and Tamil), or English; all chose English. The interviews were audio-recorded with the consent of the participants. The RA kept field notes during the data collection. The authors of this paper were not involved in participant recruitment or data collection (especially the first and third authors) as they could have been potentially known to the participants because of their professional affiliations at the time of the study.

After each interview, the RA transcribed the audio-recordings verbatim (except the names of persons and places mentioned in the interviews). Data analysis was parallel to the data collection and allowed emerging themes from early interviews to be checked and incorporated into the later interviews. Analyses of the transcribed text and field notes were based on inductive thematic analysis. This allowed data to be examined through an open ‘lens,’ and themes to emerge from the data itself [14].

The transcribed text was open coded by two authors through multiple readings. Codes were modified through discussions among the authors and by re-reading interviews and using the conceptual framework for inter-professional work (see below) [15] which provided insights into the results. Once the authors reached consensus on the coding scheme, and on the grouping of the codes, they were organized into subcategories, categories, and themes. The emerging themes were modified through discussions among the authors. To ensure trustworthiness, method triangulation (using field notes and interviews), member checks with each participant during his interview and with other participants in subsequent interviews, and peer debriefing and review were used.

### Conceptual framework for interprofessional work

Reeves and colleagues’ contingency approach to interprofessional work provides a useful framework to explore different elements of interprofessional work such as shared team identity, clear roles/goals, interdependence, integration, and shared responsibility in defining teamwork [15]. A team can have different strengths and weaknesses in terms of each of these dimensions. For example, a team can have a strong shared team identity, and at the same time, weak integrated work practices. This approach provides a complex and nuanced view of teamwork, and each team could be matched to the purpose they are intended to serve, as well as their local needs. For some purposes, shared responsibility among team members may be more important, while clear roles and responsibilities would be suitable for other purposes.

**Fig. 1:**
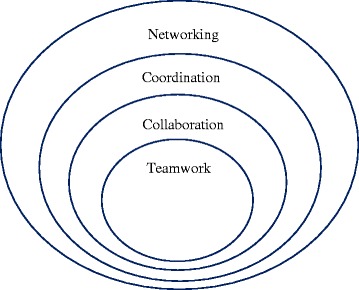
Different forms of interprofessional work. Reproduced with permission from the authors [15]

**Fig. 2:**
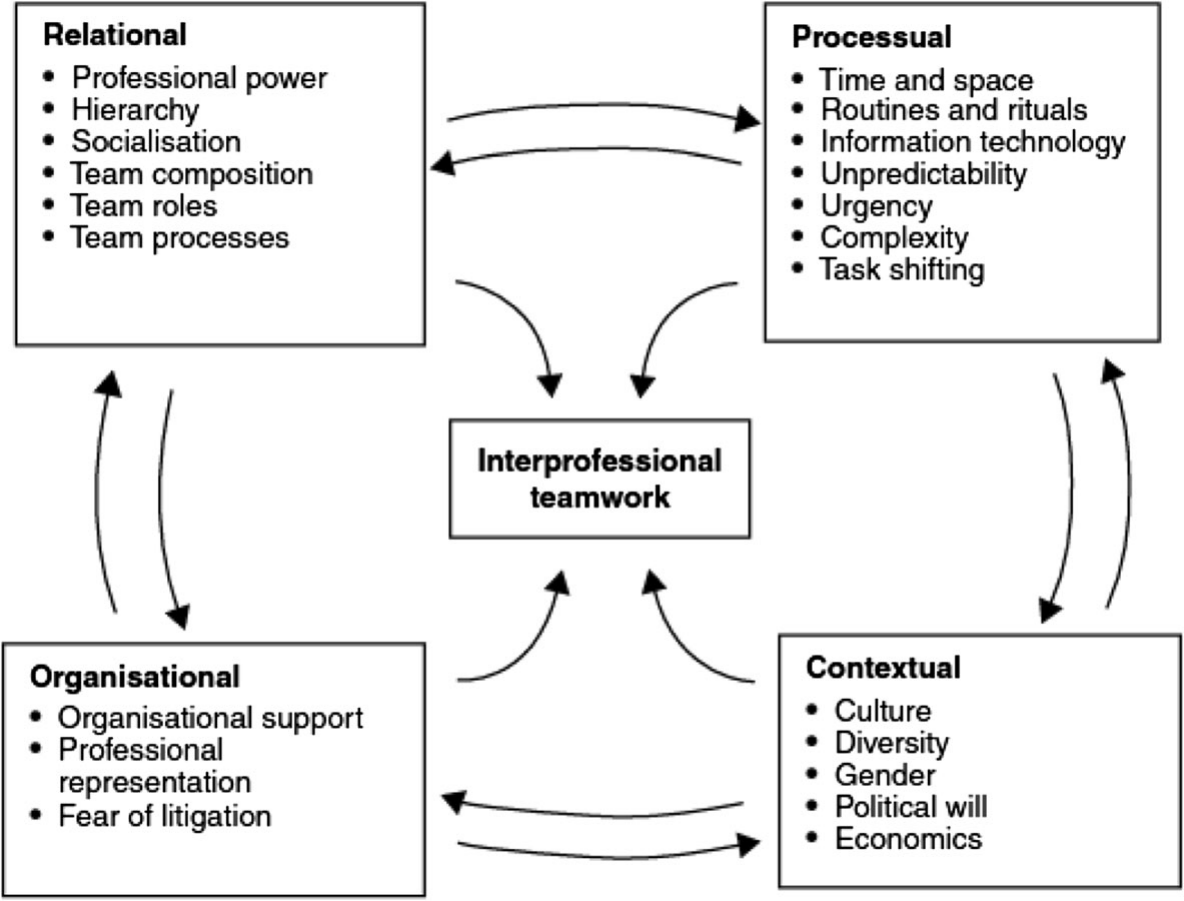
Factors related to interprofessional teamwork. Reproduced with permission from the authors [15]

According to the contingency framework, *teamwork* is only one of the different forms of interprofessional work (see Fig. 1 below), which is the most focused level of organization with high levels of interdependence, integration, and shared responsibility. Other forms of interprofessional work include, *collaboration, coordination,* and *networking*, with decreasing levels of interdependence, integration, and shared responsibility among team members at each level. According to this framework, interprofessional teamwork is influenced by various relational, organizational, processual, and contextual factors, as shown in Fig. 2 below.

## Results

### The organization of operating room work and characteristics of the sample

The study setting is one of the 8 teaching hospitals in the capital province of Sri Lanka [16]. In this hospital, similar to many other hospitals in Sri Lanka, general/routine surgical teams include one to two senior surgeons, as well as a few junior surgeons and doctors rostered from among the staff in each surgical ward/unit. For an allocated day or session in the operating room, the surgeons are joined by an anesthetist who is rostered from among the anesthetists in the hospital, independent of the surgeons’ roster. The nurses in the operating room include, one to three nurses who are directly involved in the surgical work, and one or more nursing sisters assigned to the operating room in a supervisory capacity without direct involvement in the surgical work. Supportive staff, referred to as *theatre labourers,* help with various tasks such as wheeling patients in and out of the operating room, transporting supplies, and removing soiled linen and garbage. At times, there are can be medical and nursing students in the operating room as observers.

Out of the 15 surgeons interviewed for this study, 7 belonged to the ‘senior’ rank (with 6 or more years of postgraduate surgical training and work experience), they are referred to as senior registrars and consultants, the latter being the senior-most position. The other 8 interview participants were of the ‘junior’ rank, i.e. surgical registrars with a few years of postgraduate surgical training and work experience, and interns/house officers who have not had specialized postgraduate surgical training. The consultants who participated in the study represented the 58 general surgeons appointed to the hospitals in the capital province at the time of data collection [16]. As is the case with majority of surgeons in Sri Lanka, all of those who volunteered and participated in the study were men. Most of them were 30–45 years old, except for two senior surgeons who were 45+ years old.

The results are presented in the following section as themes characterizing the nature of interprofessional work, the relational and contextual factors influencing interprofessional work, and the nature of surgeons’ non-technical skills development and training. Quotes are labeled to indicate the seniority of the respondents; senior surgeons and senior surgical registrars are identified from junior surgeons and doctors.

### Everyone would contribute, but not in an equal manner

According to the surgeons who participated in this study, a team was “*a group or a collection of members who come together for the purpose of achieving a common goal*.”

Teamwork was seen as not being limited to the surgical procedure, but as extending to the entire trajectory of the procedure, from the pre-operative to the post-operative stage. Surgeons’ conceptualization of ‘*good*’ teamwork was related to the successful surgical outcome for the patient.

Basically, the team joins for the common purpose, an operative intervention for the cure of the patient. It cannot be done individually. It has to be a collective effort of a group of people. So it’s a group of people with a common target. (Senior Surgeon ref #3)

The surgical team is a team involved in the surgery of the patient. Whether it is pre-operative, intra-operative or post-operative, to give optimum care to the patient to improve the outcome for the patient. (Junior Surgeon ref #11)

A team is a group of professionals and others who join together with different abilities and different knowledge, who work together to achieve some common goal, common purpose. (Senior Surgeon ref #1)

The different team members’ contributions were recognized as important in achieving a common goal, for example, one surgeon noted “*it cannot be done individually.”* However*,* all the surgeons perceived their role as overarching, more important, and compensatory (they see themselves as being responsible for others lapses), compared to the other healthcare professionals’ role in the team:

Everyone would get involved, but not in an equal manner. But ultimately all the responsibility is on the surgeon. Although each member’s contributions can be different, the team would achieve its goal in the end [because of the surgeons]. (Junior Surgeon ref #5)

Anesthetists’ contribution to the team was also seen as complementary to the surgeons’ role, and in some instances, they were perceived as limiting the surgeons’ role because they were not flexible or supportive:

The surgical team mainly wants to get the surgery done. But the anesthesia team [doesn’t]. They have the responsibility to [ensure] whether the patient is fit for anesthesia. There are some anesthetists, they are a bit more [excessively] careful and less flexible to allow the surgery to happen. (Junior Surgeon ref #6)

### Demarcation of roles: surgeons as leaders and other professionals as assistants

Surgeons identified and clearly demarcated the different professional groups in the team. In fact, some talked about “*the surgeons’ team* and *the nurse’s team*,” and others referred to *“the anesthetists’ team”* as a separate entity. More commonly, however, surgeons and anesthetists were grouped together and referred to as “*professionals*,” while nurses and supporting staff were referred to as “*others*.”

The categorization of team members into different professional groups as well as the demarcation of roles and responsibilities, were seen as effective team processes.

The senior surgeons were seen as having a prominent leadership role. One surgeon said *“in a team there should be a leader and some other level people to help him.”*

Nurses were considered to predominantly have an “*assisting role,”* which mainly consisted of following the instructions given by the surgeons and helping surgeons to fulfil their (more important) role.

Generally, what they do is assist our management. Really they do whatever we say. What they do is run around our instructions. (Senior Surgeon ref#3)

Nurses’ inability to fulfil this supportive role was seen as potentially causing problems; for example, a junior surgeon noted*: “when the doctor is involved in a surgery they need lots of support. Sometimes the nurses fail to give that support to the extent that the doctor wants [in this case] there are problems.” (Junior Surgeon ref#5)*

### Team hierarchy and established norms

The team hierarchy was seen as both a norm and a characteristic  of interprofessional teamwork. As one surgeon explained, “*for the last twenty to thirty years it has been like that, so that is how it’ll be for the next fifty years or so.” (Junior Surgeon ref #4).* Moreover, many of the surgeons perceived the clear and hierarchical organization as contributing to efficient team functioning, and therefore the need for such a system was justified: “*if you train a soldier he will oblige with your instructions one hundred percent. So obviously, when you’re working in a team if your junior staff member does not oblige one hundred percent in carrying out your instruction, there can be obvious problems.” (Junior Surgeon ref #12)*

The senior-most surgeon in the team was considered to be the leader and assumed the role of the primary decision-maker of the team. It was expected and accepted by the team members that he should be firm in his role; in order to be a good leader, he could not be “*too flexible*”. An important aspect  of the leader’s role was giving specific instructions or “*commands”* to junior surgeons and “*others”* on the team.

The team leader will allocate by telling the person’s name and giving the role. Each and every person will do that particular role. (Junior doctor #9)

As a leader you have to be firm. You ARE a leader. When you’re a leader you can’t be too flexible then you can’t be a good leader. (Senior Surgeon #2)

Junior surgeons valued the senior surgeons’ leadership role. They valued the leaders’ role in assuming responsibility on behalf of the team, particularly, when there were adverse events or outcomes, and in supervising and mentoring juniors in the team.

The consultant is the leader. He is like an umbrella who is looking after everything. So whatever goof thing somebody does goes back to the consultant, that’s ultimately his responsibility. (Junior Surgeon ref #4)

The senior consultants are the ones who are responsible for all the patients. Some things are done by the junior staff, but still the seniors are responsible for patient management. They are the ones who train us [the juniors] always. (Junior Surgeon ref #13)

Nurses occupied a position below that of the surgeons in this hierarchy; their main role was to comply with, and carry out, instructions swiftly and efficiently. The nurses’ role was viewed as important because it provided the surgeons with necessary support. As one surgeon reported: “*generally, what the nurses do is they assist our management” (Junior Surgeon ref #6).*

Certain team processes reinforced this hierarchy. For example, there was a preference for nurses who had prior experience with the surgeons in the team because this allowed nurses to effectively fulfil their supportive role. Working with the same team members was seen as developing familiarity with the surgeons (or their work) so that nurses could swiftly respond to the surgeon’s needs, even without them having to give any instructions.

Basically in my experience they’re [nurses] like computers. If the hierarchy gives them a command they will do it, like a computer program. Most of them will depend on their seniors to instruct them on what to do. (Senior Surgeon ref #1).

I would say the senior nurses are very experienced. Now during our surgical session, [with] a junior nurse we have to give instructions on what to do. But with a senior nurse, before we instruct what to do, they have all the instruments [prepared and ready]. It is very easy and time saving. If the nursing staff is an experienced person there is no tension. (Senior Surgeon ref # 07)

There was also a perceived hierarchy amongst the nurses. This hierarchy was based on seniority in terms of years of work, and was viewed as important for effective teamwork.

### Overstepping boundaries and potential for conflict

According to the senior surgeons, junior surgeons and nurses did not have a role in decision-making. Junior surgeons noted this as a barrier to their full participation in teamwork because, their ability to make important contributions to decision making was not recognized by the senior surgeons.

In decision making, obviously the contribution from the junior staff is very minimal. Even if the junior staff make contribution, in the final decision making they don’t make a big contribution most of the times, that’s fair enough in certain incidents. In certain situations I think it should be considered because even the stupidest question raised by the junior member, may have a valid a point. He might sometimes make a suggestion, but no one will hear that [take it into consideration]. (Junior Surgeon ref#10)

Because junior surgeons and nurses would not speak up and/or raise any concerns they may have with the senior surgeons, there was potential for communication barriers between team members. However, senior surgeons did not to see this as a barrier to communication, because according to their perception, when there was clear demarcation of roles and responsibilities, there was less of a need to ask questions.

Stepping outside of the demarcated role, particularly for the nurses, was not deemed acceptable, and was seen as potentially leading to conflicts. As one surgeon describes below, a nurse who demonstrates being more knowledgeable than a doctor (by speaking up) is seen as stepping outside of her role. If this were to happen, a surgeon would reinstate his hierarchy when the opportunity arises.

Sometimes when there are junior doctors in the theatre and nurses who may have worked there for ten to fifteen years. The nurses may know more than the doctors. If the doctor doesn’t know and the nurses know that will be an issue, then the relationship will be affected and when that doctor becomes senior he will keep that in mind and he will hold it against the nurse. That normally happens. (Junior Surgeon ref #5)

### Lack of motivation and time for non-technical skills development

Developing non-technical skills was not considered a priority by the surgeons. The reasons for this were lack of such training opportunities, and a lack of time to participate even when such training opportunities were available due to heavy workloads. In addition, surgeons also noted that there is a lack of incentive for participating in training either in terms of career advancement and/or a salary increase.

Most of the time we are attending to patients or operating or doing a lot of important activities, rather than going for training. Nobody would go. Say you had a hundred and fifty patients to see in 4 hours [in the clinic], so you can’t neglect that and go for a training. Every day is like that. In a surgeon’s life I don’t think there is enough time to get trained in those aspects. (Junior Surgeon ref #4)

I don’t blame anybody [for not getting trained], because in a setting where you are not being promoted, where you are not being rewarded for your good work, people might be hesitant about going for training. (Junior Surgeon ref#6)

Another reason for not prioritizing non-technical training was because the primary skills required for teamwork were considered to be the ability to lead others and to make decisions. According to the senior surgeons, this was a skill inherent in those who were given/held the leadership role.

Training is not important, because if you are the decision maker, if you are the main person, you know how to cope up with them, basically you can lead the surgical team towards success. (Senior Surgeon ref#1)

As such, junior surgeons were expected to learn the necessary non-technical  skills by observing and modelling senior surgeons actions; there was an expectation of being correctly guided by the seniors.

Basically we watch the seniors and if we are doing something wrong they will guide us. (Junior Surgeon ref #11)

## Discussion

This is the first study in Sri Lanka to investigate interprofessional teamwork in operating rooms using qualitative methods. Although this paper is limited to information gathered from interviews with surgeons in operating room teams from one teaching hospital, it provides useful insights into the nature of interprofessional teamwork in this setting.

The findings of this study could be strengthened by direct observation of team interactions, however, given the exploratory nature of this research and the need to ensure confidentiality for those participating, direct observation of operating room work was not possible. During informal interviews with surgeons, anesthetists, and nurses in this hospital, nurses’ involvement in the study was seen as necessary. However, as the number of surgeons, anesthetists, and nurses in the hospital is small, collecting data from all three groups at the same time was seen as potentially compromising participants’ anonymity. Therefore, anesthetists and nurses from the same operating room teams were invited to participate in the study for data collection at a later date, however, their perspectives are not included in this paper.

### The collaborative nature of interprofessional work

Surgeons’ perception of teamwork revealed a lack of shared team identity-often the surgeons referred to themselves, nurses, and anesthetists as separate (sub) teams working within the same operating room. Surgeons perceived their role in the team as more important than that of other professionals and, as a result, failed to recognize the interdependent nature of the team members’ work. Attempts to integrate their work with nurses were not described, nurses were considered to be only playing a supportive role.

Surgeons demarcated clear roles for nurses, anesthetists, and other supportive staff in the team. Having such clearly demarcated roles was considered to be one of the elements of ‘good’ teamwork; almost all of the surgeons described different team members’ ability to effectively fulfil their separate roles as contributing to efficient team functioning.

Some elements of interprofessional work as described in the conceptual framework-interdependence, integration, shared team identity, and shared responsibility [15]-appeared to be weak among the operating room teams in this setting. Demarcation of clear roles, on the other hand, was a strong element of interprofessional work. Interprofessional work appeared to be in the form of *collaboration,* a broader conceptualization than the more focussed form, i.e. teamwork (Fig. 1). The collaborative approach could be applicable because of the stable composition of the teams and the less ad-hoc nature of team formation in this setting. Stability and familiarity among team members could help teams function with less integration and interdependency, however, as discussed next, these team characteristics could also act as impediments to efficient team functioning.

In some settings, stability in team membership has been important in facilitating interprofessional collaboration, however, in others, it has also created hierarchies and communication barriers, [15, 17]. Because the number of surgeons and nurses in the hospital is small, team members tend be familiar with each other and have opportunities to form relatively stable teams over time. As described by a senior surgeon in this study, familiarity with team members could contribute to what he perceived to be efficient team functioning-because nurses familiar with the surgeon were able to better support his work. However, in contrast to other settings where stable membership helped gain mutual trust and understanding between professional groups, here it appeared to reinforce pre-existing power perceptions and help maintain rigid status hierarchies in the team. Team members’ personal history with others in the team, and their knowledge about the dispersion of power within the team, could prevent teams from perceiving underlying conflict, and addressing communication barriers. Such pre-existing power perceptions, which is the way in which team members perceive themselves in relation to their team members, can in some situations create communication barriers and veil underlying conflicts [18].

What surgeons deemed efficient team functioning was based on their own perceptions of cooperation within their teams, i.e., nurses’ and other professionals’ ability to closely follow instructions “*like computer programs,”* complete tasks without asking questions, and promptly and efficiently respond to “*commands”* or “*run around” s*urgeons’ instructions. One junior surgeon talked about the potential for tensions between nurses and doctors, however, most senior surgeons, particularly those who led the operating room teams, believed everyone was working well together. This may indicate a breakdown of communication between members, and unresolved, unspoken tensions and conflict. Surgeons are known to lack knowledge about conflict within their teams and have been reported as often failing to perceive tensions within their teams [18, 19]. This is often due to the large power differences-such as those observed here between junior and senior surgeons as well as nurses and surgeons-which could prevent those in the lower strata from feeling safe to voice an opinion or speak about their concerns. The team leader also plays an important team role in influencing and motivating other team members to speak up and play a role in decision-making [15, 18, 19]. However, team leaders who take a transactional or authoritative approach to leadership can discourage team members, including their own junior surgeons from playing a role in important team processes such as, in voicing an opinion or making decisions.

Team leadership and hierarchy have been described as important team processes in various other settings [18, 20] and this seemed to be the case here, however, there appeared to be unique relational and contextual factors, described next, that influence such team processes in this setting.

### Relational factors influencing interprofessional teamwork: Professional power, hierarchy, and socialization

A prominent status hierarchy was observed within the teams, both between and within professional groups. In other settings, team hierarchies are known to facilitate and impede teamwork [18, 21, 22]. Similar effects were also observed here; the hierarchy enabled senior surgeons to supervise junior surgeons, and by overseeing team processes and outcomes, seniors created a sense of stability and security for the juniors. However, at the same time, the hierarchical organization also disempowered juniors by limiting their participation in important team processes. Junior surgeons felt devalued because they were not contributing to an important team process, namely: decision-making. More importantly, hierarchical arrangements reinforced traditional notions of dominance, both professional and gendered forms [19, 21, 23] creating particularly prominent power gaps between the surgeons, nurses, and other team members in this setting.

Throughout the interviews, surgeons clearly demonstrated their professional power, establishing boundaries, and separating “*us”* from *“them.”* The process of identifying those that are different from one-self (i.e. othering), is a phenomenon that has been documented in healthcare settings [22, 23]. Othering can be intentionally used to reinforce and reproduce positions of domination and subordination. It is also an indication of a work culture that centers on creating valued positions for certain professions while diminishing the role and position of others. One surgeon in the study recognized that a privileged position was enjoyed by them, however, it was perceived to be the norm: “*I am not putting us high up [above others] but it’s like that.*’Although it was not perceived as deliberate othering in this setting, professional groups are known to seek a relative dominance over each other for a more privileged social and economic status. Othering is often also related to professional socialization, because when individuals seek membership in valued groups, they are expected to acquire the norms, values, and attitudes associated with that group.

One of the unique ways in which professional socialization happens in this setting, and is used to maintain a relatively higher professional status, is through the language of communication between team members. Across Sri Lanka, and in this hospital, surgeons, anesthetists, and doctors, communicate in English when conducting clinical work in the operating rooms and wards. Nurses and supportive staff would speak in the local languages (Sinhalese or Tamil) when talking to surgeons and doctors and during their own socialization. As such, language could be used to create and set professional boundaries and hierarchies, and to limit socialization within their own professional groups. This is also reflected in the surgeons’ choice of language for the interview. Although the predominately spoken language in this setting is Sinhalese, all of the surgeons preferred to be interviewed in English. Use of language for othering has been described in healthcare settings in other countries, however, this is mostly documented in relation to healthcare provider and care seeker interactions [23].

### Contextual factors influencing interprofessional teamwork: Patriarchy and gender norms

In addition to professional socialization, the gendered division of labour between male surgeons and female nurses adds another layer of complexity to interprofessional work. In Sri Lanka although more than 90 % of nurses are women, the majority of surgeons as well as those in decision-making positions in healthcare institutions such as hospital administrators tend to be men [24]. As a result the nurses’ role is always perceived as ‘women’s work’ while the surgeon’s role is perceived as work that is/can be effectively done by ‘men.’ It is noteworthy that the surgeons interviewed here always used the male pronouns “*he*” or “*him*” to refer to the team leaders.

In patriarchal societies such as Sri Lanka, gender norms dictate particular roles for women and men and these could also translate into an organizational culture that created hierarchies and subordinate positions for female healthcare workers such as nurses. As male doctors in general, and male surgeons in particular, are esteemed in society, the gender and power gap is prominent in the operating rooms, more so than in any other healthcare setting. For example, nurses, even those who have more experience and many years of service than the doctors, would address surgeons as ‘sir’ or ‘doctor.’

However, the gendered dimension of the work hierarchy and the gendered division of teamwork are not directly talked about by the surgeons, and this may be due to lack of gender diversity within the different professional groups, For example when all of the nurses are females, there would be no opportunities to observe and to talk about nurses’ work in gender specific terms. However, the gendered dimension of operating room work and the subordination of nurses is seen in the way surgeons describe them as those who “*follow 100 % of our instructions”* or as those who “*run around our instructions*.”

### Training and mentoring junior surgeons

Other studies have shown that individuals seeking membership in groups that are valued, such as professional bodies, tend to socialize exclusively with those group members, and adapt behavior consistent with the group identity [23]. A similar phenomenon was observed in this setting, particularly the junior surgeons, as they idolized seniors and the esteemed position held by them as team leaders and decision makers. As a leading teaching hospital in the country, this is particularly relevant for the training and mentoring of junior surgeons. Junior surgeons seem to derive their understanding of appropriate clinical practices by observing and modeling senior surgeons. Because of the lack of training opportunities to develop non-technical skills such as communication and team building skills, and/or lack of time and motivation to utilize the few opportunities that were are available, junior surgeons predominantly learned non-technical skills also by observation and role modelling senior surgeons. However, within the context of existing power and gender gaps, and the gender and status hierarchies, opportunities to observe positive interprofessional interactions and to develop mutual trust and respect for juniors, nurses, and other professional groups are unlikely to be available for juniors through this method of learning. As such, junior surgeons would not have opportunities to acquire skills that can help them improve interprofessional integration interdependence on each other, and to also develop a sense of shared team identity.

## Conclusions

This exploratory study provides a nuanced view of teamwork and helps to understand the strong and weak elements of interprofessional work in this setting. Various factors that directly affect the relationships shared by professionals, such as, professional power, hierarchy, and socialization, as well as contextual factors related to the broader social conditions, such as, patriarchy and gender norms influenced teamwork, created professional boundaries, and communication barriers between professional groups. As professional identities are developed through socialization and team processes are learned through role modeling and shadowing of seniors, better interprofessional integration of work is unlikely to happen without changes to doctors' and surgeons' education and training programs and the organizational structures in healthcare settings, as well as attitudinal changes towards other professional groups in healthcare teams.

In addition to these long-term, broad, systemic changes, it would be important to remove some of the existing barriers to communication within and between professional groups, in the short-term, in order to prevent adverse events in operating rooms. A formal standardized protocol, such as, the WHO Surgical Safety Checklist [25, 26], which can help bridge communication gaps and improve sharing of information within the team, could be useful for this setting, as it has proven to be in some others.

## Footnotes

1 The name of the hospital is not included here to protect the identity of the participants.
